# Opportunities and Challenges for Chinese Elderly Care Industry in Smart Environment Based on Occupants’ Needs and Preferences

**DOI:** 10.3389/fpsyg.2020.01029

**Published:** 2020-06-04

**Authors:** Qingfeng Meng, Ziming Hong, Zhen Li, Xin Hu, Weixiang Shi, Jun Wang, Kai Luo

**Affiliations:** ^1^School of Management, Jiangsu University, Zhenjiang, China; ^2^School of Architecture and Built Environment, Deakin University, Geelong, VIC, Australia; ^3^Australasian Joint Research Centre for Building Information Modelling, School of Built Environment, Curtin University, Bentley, WA, Australia

**Keywords:** smart elderly care, intelligent environment, opportunities and challenges, news reports, content analysis

## Abstract

New developments in intelligent devices for assisting elderly people can provide elders with friendly, mutual, and personalized interactions. Since the intelligent devices should continually make an important contribution to the smart elderly care industry, smart services or policies for the elders are recently provided by a large number of government programs in China. At present, the smart elderly care industry in China has attracted numerous investors’ attention, but the smart elderly care industry in China is still at the beginning stage. Though there are great opportunities in the market, many challenges and limitations still need to be solved. This study analyzes 198 news reports about opportunities and challenges in the smart elderly care industry from six major Chinese portals. The analysis is mainly based on needs assessment for elderly people, service providers, and the Chinese government. It is concluded that smart elderly care services satisfy the elders’ mental wants and that needs for improving modernization services are the most frequently mentioned opportunities. Also, the frequently mentioned challenges behind opportunities are intelligent products not being able to solve the just-needed, user-consumption concept and the ability to pay, which is the most frequently mentioned challenge. The results of this study will enable stakeholders in the smart elderly care industry to clarify the opportunities and challenges faced by smart elderly care services in China’s development process and provide a theoretical basis for better decision making.

## Introduction

At present, China has become the country with the largest number of elderly people in the world. According to the latest statistics from the National Bureau of Statistics, the number of elderly people over the age of 60 in China has been increasing in recent years. In 2013, it exceeded 200 million, accounting for only 14.9%, and it reached 240.9 million in 2017, breaking the record by 17%. By the end of 2018, the population aged 60 and over was 249.49 million, accounting for 17.9%. Among them, the population aged 65 and over was 166.58 million, accounting for 11.9%. Compared with that at the end of 2017, the proportion of the elderly population continues to rise. With the aging of the population, the state has paid more and more attention to the development of the old-age industry, issued a series of policy support, gradually improved the social security system for the elderly population, fully opened the development of the aged care service market, vigorously prospered the old consumer market, and actively promoted the community-based in-home elderly care, the combination of medical care and elderly care and other models.

The smart elderly care industry is a product under the background of the “supply-side structural reform” and a recipe for solving the imbalance of China’s elderly care industry structure. Smart health and elderly care refer to the integration of information technology and products such as health care electronics, Internet of Things, cloud computing, big data, and mobile Internet for collecting data such as human signs and home environment; realizing family, community medical institutions, health care services and information interconnection, and analytical processing between professional medical institutions; and providing intelligent, personalized, and diversified products and services to meet the increasingly urgent health needs of the people (Yang et al., 2016).

Wisdom for the elderly plays an important role in improving the quality of life of the elderly and has attracted the attention of many investors and government departments. The smart elderly care industry grows rapidly since the state council published the guideline for promoting the development of “Internet+” in July of 2015. The Premier of the state council pointed out that the smart elderly care industry needs to be improved as soon as possible, in the Report on the work of the Chinese government. In the early 21st century, the smart elderly care service was employed in the United States and European developed countries, and now it is in a mature stage. For example, as early as 2007, Europe states established the Active and Assisted Living (AAL) Research and Development Program to promote the smart elderly care industry (Courtney et al., 2008; Panek and Zagler, 2008). Over the last 10 years, the AAL program has focused on improving the well-being of older adults through the use of adapted digital technology. The AAL forum also attracts potential investors and buyers looking for commercially viable solutions (Cotten et al., 2016; Siegel and Dorner, 2017). Since 2008, the smart elderly care industry in the United States has been committed to bridging the digital divide between technology and health care so that people can live healthier lives. However, the development of China’s smart elderly care system is still in its infancy, and many factors are affecting the development of the smart elderly care industry, such as the residents’ needs and preferences and the service level of product service providers. Some of these factors may positively promote the development of a smart elderly care environment, and some may become obstacles in the development of the smart elderly care industry.

To more effectively promote the healthy and sustainable development of the smart elderly care industry in China, we need to clarify the opportunities and challenges faced by the smart elderly care industry in China’s development process. First, the domestic industry practitioners are keen to understand these opportunities and challenges to formulate effective property management strategies, which is crucial for them to obtain competitive opportunities especially in the initial stages of the industry development. This will also guide the development of China’s smart elderly care market. The development of China’s smart elderly care system is still in its infancy, and many factors are affecting the development of the smart old-age environment, such as the residents’ needs and preferences and the service level of product service providers. Some of these factors may positively promote the development of a smart elderly care industry, and some may become obstacles in the development of the smart elderly care industry. With the aging of the population, China’s government has paid more attention to the development of the smart elderly care industry. The government has published a series of policies that support the industry: “Deeply develop the elderly care industry,” “Deeply promote innovation-driven development strategy,” and “Deeply integrate “Internet+” and smart elderly care industry.” The smart elderly care industry in China is just beginning to burst, so there are lots of opportunities and challenges in the industry. Therefore, it is very important to find out all opportunities and challenges in the smart elderly care industry of China overall. The findings in this paper will guide the industry in the right direction.

This paper uses content analysis methods to analyze the news reports about the smart elderly care system from June 2018 to June 2019. It comprehensively summarizes and analyzes the opportunities and challenges of China’s smart old-age environment in the development process. The research findings will not only provide valuable implications to the industry practitioners but also help the government formulate appropriate strategic property management strategies to ensure the healthy development of this sector.

The chapters of this paper are arranged as follows: the second chapter will summarize and analyze the current status of China’s smart elderly care; the third chapter introduces the research methods of this paper; the fourth chapter summarizes the opportunities and challenges that we can understand through the analysis of China’s smart old-age environment in the development process; the fifth chapter analyzes the existing obstacles and gives corresponding countermeasures and suggestions; and the sixth chapter is the summary of this paper.

## Analysis Of The Current Situation Of Smart Elderly Care In China

This study focuses on smart care service for Chinese elderly people. This analysis exposes a better understanding of the most popular topics in the smart elderly care industry, including the status of the elderly care service and smart community of elderly people in China. By depicting a clear picture of related topics, the theoretical background of this study is provided.

First of all, for the old-age care and old-age environment of the elderly in China, aging at home, usually co-residing with family members, is the most popular living arrangement for older adults in China (Silverstein et al., 2006). The conventional Chinese cultural pattern of filial piety, which obliges family members to assist their elders, has contributed to the close connections between different generations (Mao and Chi, 2011). The Chinese government has also enacted laws (e.g., the Elders’ Protection Law) to maintain this traditional family support system to care for older people financially, physically, and emotionally (Wu et al., 2005). In China’s small-scale peasant society, the family is the foundation of society and is the most basic unit. At present, with the continuous development of the economy and the continuous improvement of people’s living standards, more and more elderly people choose to support the elderly in some old-age communities. With the further development of the market economy, social changes, and Chinese-style “family care” model, a new model consisting of “community care” and “institution care” has been produced (Zhu et al., 2014).

Secondly, with the gradual maturity of technologies such as artificial intelligence and big data, the industry tends to be informal, convenient, and intelligent. For a time, those who are entering are talking about “intelligence and old age,” and more technology and energy are left in old age. On the smart hardware, the design and use of smart devices in China has emerged in China’s old-age community. Regarding the *status quo* of China’s smart elderly care, China is changing from an agricultural society to an informational society to achieve the “modernization” found in the Western world (Economy, 1998). With the rapid development of information and technology, the smart retirement community is also a fast-growing thing. Moen and Erickson (2001) designed the smart elderly community as an age-homogenization facility that provides residents with a variety of services and facilities to meet their changing needs in an environment. Jenkins et al. (2002) suggest that the smart elderly community life can benefit residents by improving health status and social interaction levels. In 2012, China began to implement the concept of a “smart city” which focused on the care of the elderly using technology (Heng et al., 2014). In 2017, the three ministries and commissions have announced two demonstrations of smart health elderly care application pilots. At present, there are 79 smart health elderly care demonstration enterprises, 130 smart health elderly care demonstration streets (townships), and 29 smart health elderly care demonstration bases.

Besides, some scholars have analyzed the motivations for the development of smart old-age care. Chou (2010) used the first random sample from large countries, guided by a comprehensive model, and used a study of urban–rural comparison methods to study the willingness of 20,255 elderly people to go to elderly care and found that in urban and rural areas, only 20 and 17% of seniors are willing to do so. For both economic and sociocultural reasons, researchers have pointed to the need to offer more support for family care and to expand and strengthen non-institutional forms of long-term care, such as home health and community-based care. This study has provided additional evidence of the need to build a long-term care system that is consistent with China’s economic and sociocultural conditions and that meets older adults’ needs and preferences. Hu (2010) relied on the demographic structure to analyze the aging trend and, by comparing with the foreign treatment methods, determined the necessity of establishing an intelligent community in China. He proposed that the intelligent elderly community can not only meet the ideal status of the elderly in the country and reduce the burden on workers but also point out a new feasible way for aging China. Therefore, it is very important to establish a Chinese smart elderly community.

The existing research on opportunities and challenges for smart elderly care industry in other countries is mainly on smart technologies and smart equipment. Habib et al. (2014) introduced the challenges and open issues of smartphone-based solutions for fall detection and prevention. Suryadevara and Mukhopadhyay (2012) introduced a wireless sensor network-based home monitoring system for wellness determination of the elderly and analyzed the opportunities of a wireless sensor network in the smart elderly care industry. Geman et al. (2015) presented some challenges and opportunities in Ambient Assisted Living (AAL) for disabled and elderly people addressing the various state-of-the-art and recent approaches particularly in artificial intelligence, biomedical engineering, and body sensor networking. Foreign researchers have less research on opportunities and challenges for the development of smart elderly care industry. So it is of great significance to find out the opportunities and challenges for the smart elderly care industry.

## Research Methods

News coverage contains valuable information, and analyzing news coverage provides a feasible way of exploring the various aspects of a phenomenon through excavating the story behind it and the perception of field experts (An and Gower, 2009). This method can clearly understand the attitudes of frontline personnel and related practitioners to the research objects and evaluate the news content as professional journalists and also explore the complex interrelationship between a thing and its complicated use environment. This method has been applied to many fields such as the humanities and social sciences.

This paper uses quantitative analysis and qualitative analysis research methods to explore the opportunities and challenges of smart old-age development in China from news reports. There are several reasons for using this method. First of all, because smart elderly care is used in the life of the elderly, it is an important development direction for China to enter the Internet of Things and the “Internet+” era, and it is also one of the specific applications of the continuous development and progress of artificial intelligence (AI). This has led to a large number of reports from the mass media in the country about this news. According to the Code of Ethics of the Society of Professional Journalists, news coverage is accurate, fair, and thorough. A careful study of relevant news reports found that most of the articles reported were some annual smart old-age-related seminars, on-site interviews organized by smart old-age care communities, and on-the-spot interviews with smart equipment manufacturers. The interviewees were also related industries’ professionals (government officials, academics, elderly users, service operators, product developers, etc.). Therefore, the information reported in the news reports used to explore the challenges of smart elderly care development in China is credible and valuable. Besides, the use of traditional survey methods (interviews or questionnaires) takes a lot of time and effort and is not efficient. As China’s smart elderly care system is still in its infancy, the use of smart elderly care products in old-age services is insufficient, the elderly are less likely to accept new things in terms of learning acceptance, and the number of professional staff engaged in elderly care smart equipment services is small. The direct interviews with direct workers took a lot of time and effort and were very inefficient. On the contrary, the use of content analysis to analyze news reports can obtain results more objectively and reliably, and it takes less time and resources and is more efficient. Also, since the development of China’s smart old-age care is in its infancy, news reporting research on this issue is very time sensitive and insufficient in research and exploration. Therefore, based on the comprehensive analysis of news reports, it is possible to further explore through interviews, questionnaires, etc., providing valuable value for the entire study. Analyzing news reports has been used in several published studies, confirming its usefulness and robustness in understanding certain issues.

**FIGURE 1:**
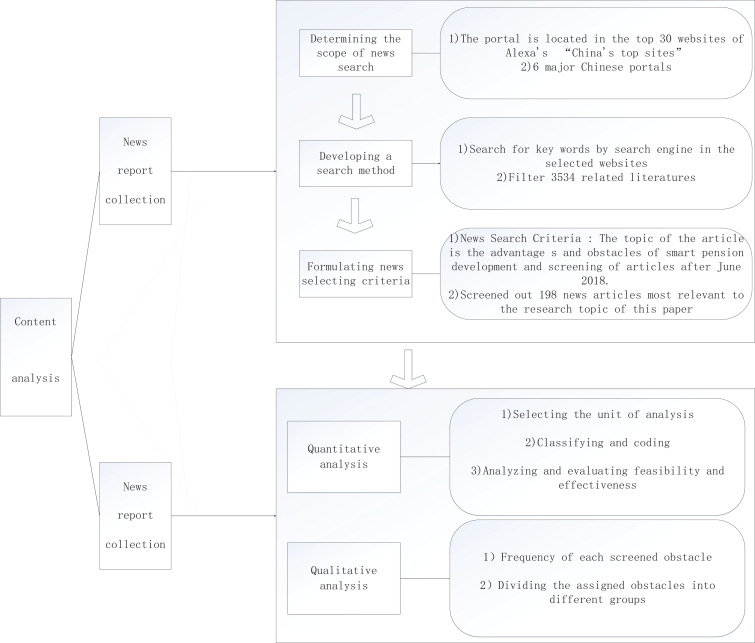
Content analysis process diagram.

Content analysis is an approach often used to analyze news coverage (Calloway et al., 2006; Qu et al., 2009). It was also used in this research to gain insights from the identified news reports. It is a systematic and objective research method for making valid inferences from collected data to describe and quantify specific phenomena (Downe-Wamboldt, 1992). Its suitability to analyze news coverage has been confirmed in the historical studies of An and Gower (2009), for example. Compared with traditional methods (e.g., interviews and questionnaire surveys), the content analysis provides more objective and reliable results (based on real and “mute” evidence). Meanwhile, it consumes less time and resources (Hu et al., 2017). Nimrod (2009) analyzed benefits to elders in the elderly community by using newspaper coverage analysis and content analysis. Mercille (2014) examined the mainstream media coverage to explore the housing bubble issue in Ireland. The successful explorations of these related studies indicated that news coverage analysis is suitable for such papers. Data collection (identification and collection of news reports on the development opportunities and challenges of smart aging in China) and data analysis (qualitative analysis and quantitative analysis of collected news reports) are two key steps, as shown in Figure 1.

The news gathering process includes the following:

### Determine the Source of News Reports

The Internet provides a convenient way to get news stories. It has the advantages of large news reports, high keyword search efficiency, and low search cost. Since most of the news reports about China’s only old-age community have been published or reprinted on major portals, the news reports used in this study are all from China’s major portals. Other sources (e.g., newspapers and magazines) were not used as it is too inefficient to collect and search related news coverage in this way and as they often involve access charges (Hu et al., 2019).

The list of “Top Sites in China” released by the Alexa web ranking service provider facilitated the identification of the major Chinese portal websites used in this study (Alexa, 2019). Alexa lists the top sites in each country/territory, and its reliability and usefulness are well accepted (Jowkar and Didegah, 2010). This study screened the top 30 websites ranked by the Chinese Alexa website in 2019. Since some websites are special, websites such as shopping websites and video websites, it is impossible to find suitable news reports. Therefore, we have filtered six portals to more accurately search for the news reports corresponding to the research content (Table 1). These websites are news websites, or their news reports are one of the important parts of their websites.

**TABLE 1:** Six portals in the news search area and the number of news selected.

**Code**	**Website**	**Rank in Alexa list (2019)**	**Number of news reports**
1	Qq.com	1	667
2	Sohu.com	4	532
3	Sina.com	6	575
4	163.com	12	424
5	Xinhuanet.com	21	697
6	gmw.cn	29	629

### Retrieving News Reports About China’s Smart Retirement Community

As shown in Figure 1, the article search is performed using a search engine in the selected portal to control the search scope to each portal. Since most news reports are in Chinese, the keywords searched are in Chinese. There are many different keywords in the news report that can represent the same meaning, such as smart elderly care, smart elderly care, high-tech elderly care, opportunities, challenges, obstacles, malpractices, and deficiencies. To get a comprehensive search result, from April to July 2019, we used multiple Chinese phrases to search on each portal. For example, we use “Smart Aging Opportunity” and “Smart Elderly care Advantages” as keywords to search for news reports on the sina.com website or the “Smart Care Challenge” as a keyword to search for news reports on the 163.com website. A total of 3,534 articles (Table 1) were obtained.

### Formulate Selection Criteria and Choose News Reports

We develop selection criteria for news stories to determine which news stories can be used. To be objective, we select 200 latest news reports on the smart elderly care industry. The articles reported are some annual smart old-age-related seminars, on-site interviews organized by smart old-age care communities, and on-the-spot interviews with smart equipment manufacturers. The interviewees are also related industries’ professionals (government officials, academics, elderly users, service operators, product developers, etc.). First, we determine the similarity between the article and the topic under study by evaluating the topic and content of the news. Secondly, considering that China’s smart devices are in the development stage, the product update progress is very fast, so we only select news reports in the past year. Thirdly, the articles selected must focus on an identified obstacle or opportunities and must mention three or more factors of obstacles and opportunities in the development of the industry. Besides, there are duplicate articles between different portals that need to be screened to ensure the quality of the selected news stories and the accuracy of the analytical data. Finally, in the 3,534 news reports, 198 news reports most relevant to the research topic were screened out. After several discussions, we finally choose 198 news reports out of the original 200 because two of them do not meet our standard. There were 58 in 2018 and 140 in 2019. They are seminars and presentations on the stakeholders of smart devices, interview articles on actual use of customers, and the development of smart old-age communities in China.

Qualitative analysis and quantitative analysis were used. Qualitative analysis involves identification and coding, which divides the barrier into different groups. This process includes the selection of analysis units, coding, grouping by category, analysis, and credibility assessment. First, the analysis unit is the basis for reporting analyses, and the most appropriate analysis unit is whole interviews or observational protocols (Graneheim and Lundman, 2004). In this study, the analysis unit is the content sentence mentioned in each article about “Smart Care for the Elderly” and “Smart Care for the Elderly.” Second, coding and grouping categories can be conducted based on either predetermined systems/frameworks or analysis of collected data. Since different news reports have different words describing the same opportunity or challenge, based on similar analysis units, we will summarize and encode a short sentence. The analysis units with the same meaning are assigned to the unified obstacle group, and the identified obstacle groups are further divided into different categories. Third, we use Microsoft Excel to build a database and enter the barriers and categories identified by the barriers. Therefore, this process can modify inappropriate opportunities and challenges group names and groupings. Based on the qualitative analysis, quantitative analysis is used to count the number of obstacle groups, and the frequency with which the obstacles are mentioned is calculated.

## Results

As shown in Tables 2, 3, 198 news reports in this paper are coded according to the form of “Category – opportunities and challenges–unit of analysis,” and the content analysis unit is classified, to cover all the contents of the news reports, and finally from the content analysis unit coding table based on the news report. Because of the article space limitation, the table does not show the full content analysis unit and coding results.

**TABLE 2:** The content analysis coding table of opportunities in the industry.

**No.**	**Category**	**Code**	**Opportunities**	**Content analysis unit**
I	Elderly user needs	1	Meet the spiritual needs of the elderly	Release loneliness issues in the elders living alone
				Satisfy diversity demands of elders
				……
		2	Meet the health needs of the elderly	Make personalized guidelines of health management
				Fast emergency response to elders
				……
		…	……	……
II	Product service offering	6	Improve the level of institutional modernization services	Improve the quality of elderly care system
				Improve the efficiency of management overall in the industry.
				……
		7	Easy management of the service organization	Achieve monitoring of the staff in real time
				Improve service efficiency in the institution
				……
		…	……	……
III	Government promotion	12	Support for government decision making	Make policies of government grants based on data collected by the smart platform
				Integrate big data analysis with a smart platform for elderly care system to make related policies
				……
		13	Ease of government regulation	Supervise the institution with the smart platform
				The government can get all data for supervising the institutions
				……
		…	……	……

**TABLE 3:** The content analysis coding table of challenges in the industry.

**No.**	**Category**	**Code**	**Challenges**	**Content analysis unit**
I	Elderly user needs	1	Insufficient market education; the elderly have a vague concept of smart elderly care	The understanding of smart elderly care service of elders is not enough
				Elders cannot accept intelligent devices due to limited education
				……
		2	Older users have poor acceptability	The intelligent device is too expensive to consume for elders
				Elders cannot afford smart elderly care service
				……
		…	……	……
II	Product service offering	5	Smart product service is not practical and cannot solve just-needed concepts	Many products in the industry still need to be improved
				Many wearable devices are not practical
				……
		6	The supply chain of services is long and fragmented, the innovation system is fragmented, and effective integration is insufficient	The overall planning in cities is insufficient
				The resource integration in the smart system is insufficient
				……
		…	……	……
III	Government promotion	14	Limited government supervision of the Smart Elderly Healthcare project development	The smart elderly care system is not comprehensive
				The policies supporting the smart elderly care industry from the local government are insufficient
				……
		15	Existing policy constraints	The relationship between the government and industry is not clear
				The relationship between the government and society is not clear
				……
		…	……	……

Table 4 shows the 14 opportunities identified and their frequencies based on the content analysis. Among them, the highest frequency is to improve the level of institutional modernization services, accounting for 59.1%. For the elderly, the main opportunity for smart retirement is to meet the spiritual and health needs of the elderly, accounting for 56.8%. The satisfaction of physiological needs and safety needs is relatively low.

**TABLE 4:** Opportunities for Chinese elderly care industry in the smart environment.

**Category**	**Code**	**Opportunities**	**Frequency (%)**
Elderly user needs	1	Meet the spiritual needs of the elderly	56.8
	2	Meeting the health needs of the elderly	56.8
	3	Meeting the physiological needs of the elderly	40.9
	4	Meeting the safety needs of the elderly	22.7
	5	Convenient to communicate with children	13.6
Product service offering	6	Improve the level of institutional modernization services	59.1
	7	The service organization is easy to manage	22.7
	8	Improve service information integration	20.5
	9	Provide precision and customized services	20.5
	10	Improve service efficiency	15.9
	11	Reduce service costs	13.6
Government promotion	12	Support for government decision making	13.6
	13	Easy for government regulation	6.8
	14	Promote the positive development of the old-age cause	4.5

Table 5 shows the 17 challenges identified and their frequencies based on the content analysis. Among them, the most frequent challenge is that smart product services are not practical and cannot solve just-needed concepts, accounting for 59.8%. The elderly have insufficient market education and have a vague concept of smart elderly care, a conservative concept of consumer consumption, and a limited ability to pay. These two issues are also relatively high, both above 30%. However, existing policy constraints, lack of policy support, lack of a unified administrative agency, and other challenges occur less frequently.

**TABLE 5:** Challenges for the Chinese elderly care industry in the smart environment.

**Category**	**Code**	**Challenges**	**Frequency (%)**
Elderly user needs	1	Insufficient market education; the elderly have a vague concept of smart elderly care	36.7
	2	Older users have poor acceptability	33.5
	3	The user’s consumption concept and the ability to pay are conservative	31.2
	4	Elders are not interested in complex smart devices	19.6
Product service offering	5	Smart product service is not practical and cannot solve just-needed concepts	59.8
	6	The supply chain of services is long and fragmented, the innovation system is fragmented, and effective integration is insufficient	35.8
	7	The industry lacks professional talents	31.7
	8	Technical stability, reliability, and applicability still need to be improved	28.9
	9	Product development is difficult and costly	23.2
	10	Wisdom for the elderly has a public interest	23.1
	11	Local community execution is weak	18.8
	12	There are immature profit patterns	18.6
	13	The business model is immature	14.2
Government promotion	14	There is limited government supervision of the Smart Elderly Healthcare project development	22.4
	15	There are existing policy constraints and lack of policy support	20.7
	16	There is a gap between policy formulation and implementation	18.7
	17	There is a lack of a unified administrative agency	17.4

These opportunities and challenges are divided into three categories: senior user needs, product service delivery, and government promotion. In terms of opportunities, the opportunities for smart retirement communities mainly face the needs of elderly users and the provision of products and services. Among the 14 opportunities, 11 are related to the needs of elderly users and product services. On the challenge side, 9 of the 17 challenges were related to product service, accounting for 52.9%.

## Discussion

Through statistical analysis of the collected news reports, we identify the opportunities and challenges in the smart elderly industry in China. The following discussion will be based on three dimensions, including elderly user demand, product service provision, and government promotion, to analyze the challenges and opportunities.

### The Dimension of Elderly User Demand

Satisfying the spiritual needs and the health needs of elders is the most important opportunity that the smart elderly care industry faces in the development of China. In the meantime, insufficient market education, the vague concept of the smart elderly care industry, poor acceptance to new things, negative consumer behavior, and the issues of ability to pay are the most important challenges that the smart elderly care industry faces in the development of China. There is enough evidence that the smart elderly care system can improve the quality of elders’ life by using advanced technologies, such as health monitoring device, elderly care devices, remote medical treatment, and non-infectious chronic disease (NCD) management cloud platform. On the other hand, the smart elderly care system can satisfy mental needs with VR/AR technology and remote care system. Intelligent devices can provide personalized interaction and service to release loneliness of elders who have no children compared to the traditional elderly care system.

Serviceability of smart elderly care products needs to be improved, though responses and reviews of smart elderly care products are quite good. Furthermore, elders still have a lot of trouble in using these products. Compared to the young generation, elders show a negative attitude to new things and have a relatively poor learning ability. So their willingness to accept smart elderly care products is not strong. Besides, smart elderly care products are expensive in general while the consumption behavior of most elders is relatively conservative. There are conflicts between the consumption behavior of most elders and the high price of smart elderly care products. Therefore, it results in many challenges in the smart elderly care industry. The manufacturers in China nationally promote the concept of smart elderly care and provide elders with a free trial to motivate the willingness of elders. In the process of national promotion, the initially targeted elders are suggested to be the elders who already have the willingness to accept intelligent devices, and then the products can be promoted within the elders’ community with positive responses and reviews. At the same time, the manufacturers need to create and update smart elderly care products and service for families, communities, and institutions, such as wearable devices for health management system and health monitoring system and robots for home services or institution services. The manufacturers can also provide rental service in some pilot cities for elders who want to experience the products but have relatively limited ability to afford the products.

### The Dimension of Product Service Offering

Improving the modernization of elderly care service and the efficiency of management in the elderly care system is the most important opportunities in the development of the smart elderly care industry. The following are the most important challenges for the industry: The smart products are not practical in many cases, the supply chain is long and fragmented, the innovation system is old, and there is a lack of effective integration in the industry. There is no doubt that smart devices improve the quality of elderly care service through the use of lots of advanced technologies, such as big data, AI, 5G, and Internet of Things. They help elderly care services to improve accuracy and efficiency. Besides, they also assist elderly care institutions by deeply finding out the demands of their customers through collecting data and analyzing the data collected by smart devices. Analyzed data will assist managers in the institutions to make the right decisions.

However, the smart elderly care industry still faces lots of problems that need to be resolved. Firstly, many smart products provided by elderly care institutions cannot satisfy the personalized demands of elders because the products still need lots of behavior data of elders to be trained. Secondly, many smart products still have trouble in data connection because manufacturers use different interface standards. So it is very hard to effectively use and share the data. The smart elderly care industry involves the IT industry, medical treatment industry, health industry, and elderly care industry. So it is urgent to set up an open standard to link those industries. The industry still has lots of issues on medical device access, medical and health data security, and development of integration of medical institutions and care institutions. Most elderly care institutions are not willing to cooperate with manufacturers in daily health management and NCD management. The manufacturers should investigate elders’ demand before developing smart products, and the surveys should categorize and divide these products into as many units. Smart products should be updated and modified through a full life cycle. The manufacturers should carefully consider elders’ willingness, the ability of acceptance, and affordability because the purpose of smart products is to improve the quality of elders’ life. The industry needs to set up several pilot smart elderly care communities or institutions to gain more experience in a practical environment. In the next few years, the smart elderly care system should be involved in smart cities to resolve Information Isolated Island issues. All the data from the medical system, elderly care system, and other systems should be connected in an efficient way to support the smart elderly care system.

### The Dimension of Government Promotion

Assisting China’s government to make scientific decisions and supporting China’s government to supervise the elderly care institutions are the most important opportunities in the development of the smart elderly care industry. The existing policy constraints are the most important challenges in the development of the smart elderly care industry. China’s government has announced the “Action Plan for The Development of Smart Elderly Care Industry” (2017–2020). In the action plan, it is mentioned that the industry should fundamentally grow to a comprehensive industry system and produce several smart elderly care brands which are impressed in elders. However, in reality, smart elderly care systems are difficult to promote in most cities in China because the action plan is too general to execute in cities of China. Many constraints and limitations in policies and regulations need to be resolved soon in every province of China. In the meantime, the smart elderly care industry is still in the beginning stage, and so China’s government just selects several developed cities to promote the smart elderly care system. In other words, it is not a real nationwide promotion in China. Lack of policies and regulations for the local government reduces the motivation of the local government for promoting the industry. So it is highly recommended that China’s government should promulgate adequate policies and regulations for the smart elderly care industry. Besides, standards and guidelines for the industry are also foundations for promoting the smart elderly care system.

In the future, the Chinese government will continue to vigorously strengthen the development of informalization around the state. The government will suggest that smart elderly care institutions need to provide elders with smart products for improving the quality of smart elderly care. The industry will be partly subsidized by the government for promoting smart products. The manufacturers and service providers need to fund a forum for bringing the government, manufacturers, and service providers together around the state to find out smart solutions and to engage with other people in the market. The forum is suggested to be cofinanced by the government and manufacturers. The specific aim is to foster the emergence of manufacturers, service providers, and systems for elders living well at home and in institutions. The elderly users should provide more reviews on smart products while enjoying the smart service to support the development of the smart elderly care industry in China. Each year, the forum issues a call designed to solve the negative reviews which help promote the elders to be positive on smart products and a call designed to reflect the opportunities and challenges in the development process of the smart elderly care industry. Through our cooperation, the smart elderly care industry in China will be on the right track.

## Conclusion

Whether it is from the top design, rigid demand, or scientific and practical convenience, smart elderly care will be an important force in the future elderly care industry. At present, there is a lot of challenges and shortcomings in the initial stage of the development of the smart old-age environment in China, bringing various opportunities. It is of great significance to clarify these opportunities and shortcomings to promote the development of China’s smart elderly care.

This paper mainly analyzes 198 news reports of China’s six major portals, explores the opportunities and challenges of smart elderly care development in China, and identifies 14 opportunities and 17 challenges. These opportunities and challenges are divided into three categories: senior user needs, product service delivery, and government promotion. According to the frequency of opportunities and challenges reported in the new coverage analysis, the most significant advantage relating to these three groups is to improve the level of institutional modernization services, and the most significant barrier relating to these three groups is that smart product service is not practical and cannot be used to solve just-needed concepts.

The research results of this paper are beneficial to China’s smart aged care service providers and China’s smart old-age care industry in many aspects. First, the domestic industry practitioners are keen to understand these opportunities and challenges to formulate effective property management strategies, which is crucial for them to obtain competitive opportunities especially in the initial stages of the industry development. Besides, this will also guide the development of China’s smart elderly care market.

Future research, such as case studies, interviews, and questionnaire survey, is needed to examine individual barriers more closely and develop practical means for their amelioration and elimination.

## Data Availability Statement

All datasets generated for this study are included in the article/supplementary material.

## Author Contributions

QM, XH, and JW devised the project, the main conceptual ideas, and proof outline. QM, ZH, and WS developed the theory and analyzed the current situation of smart pension in China. ZL and KL verified the analytical methods. QM and XH encouraged ZH and WS to investigate quantitative analysis and qualitative analysis research methods. QM and ZL supervised the findings of this work. All authors discussed the results and contributed to the final manuscript.

## Conflict of Interest

The authors declare that the research was conducted in the absence of any commercial or financial relationships that could be construed as a potential conflict of interest.
